# To each their own: a review of individual differences and metaphorical perspectives on time

**DOI:** 10.3389/fpsyg.2023.1213719

**Published:** 2023-08-10

**Authors:** Michele I. Feist, Sarah E. Duffy

**Affiliations:** ^1^Department of English, University of Louisiana at Lafayette, Lafayette, LA, United States; ^2^Department of Humanities, Northumbria University, Newcastle upon Tyne, United Kingdom

**Keywords:** time, metaphor, individual differences, Moving Time, Moving Ego

## Abstract

How do people talk—and potentially think—about abstract concepts? Supported by abundant linguistic evidence, Conceptual Metaphor Theory posits that people draw upon concrete concepts to structure abstract ones via metaphorical connections. Often, the source domain for a metaphor draws upon embodied physical experience, as in the time is space system, whereby representations in the domain of time are thought to arise from experiences of navigating through, orienting within, and observing motion in space. In recent years, psychological evidence has suggested that the connections between space and time are indeed conceptual; however, many gaps in our understanding of the workings of metaphor remain. Notably, until recently, the unique variations in the ways in which people experience metaphor have been largely overlooked, with much research falling prey to what Dąbrowska has identified as one of the ‘deadly sins’ of cognitive linguistics: to ignore individual differences. By focusing on two widely studied metaphors for time, Moving Time and Moving Ego, this review article shines a spotlight on the varied ways in which people draw on their embodied and enculturated experiences, along with ‘human experience’ on an individual level and the contexts within which they use metaphor. In doing so, it highlights the importance for metaphoric conceptualization of variation across languages, across contexts, and across individuals, suggesting that while the use and interpretation of metaphor may begin with cross-domain connections, they are but part of the story.

## 1. Introduction

Across recent decades the question of how people represent and reason about that which we cannot perceive directly through the senses has been of central interest to scholars working in the cognitive sciences (Markman, 1999; Gentner and Bowdle, 2008; Bolognesi and Steen, 2018). One prominent proposal, Conceptual Metaphor Theory (CMT), holds that abstract concepts are understood in terms of more concrete concepts, which are more closely connected to everyday physical and perceptual experiences and, hence, more easily reasoned about (Lakoff and Johnson, 1980). Abundant evidence for this proposal comes from linguistic metaphors, in which abstract concepts are described in terms of more concrete ones, often drawing upon correlations in our embodied physical experience (Grady, 1997). For example, the correlation between physical motion toward an object and our expectation of being located with the object in the future gives rise to the conceptual metaphor time is (motion along) a path (Grady, 1997, p. 119), motivating expressions such as “We’ve *arrived at* the moment of truth.” In a parallel fashion, motion of an object toward ourselves correlates with a progressive shortening of the time until it is at our location, giving rise to the conceptual metaphor time is the motion of objects (Grady, 1997, p. 119), underlying expressions such as “Spring is *coming*.” Through metaphorical connections such as these, our sensory-motor experiences and our interactions with people, objects, and the world at large may provide a basis for understanding many aspects of life that are not perceived through our senses (*cf.*, Rosch et al., 1991).

The physical realities of our spatial world permeate our daily lives, and metaphors drawing upon the domain of space abound (Clark, 1973; Haspelmath, 1997; Lakoff and Johnson, 1999). Indeed, Lakoff and Johnson (1980) argue that “most of our fundamental concepts are organized in terms of one or more spatialization metaphors” (p. 17), meaning that much of how we think and understand our world can be structured by our experiences in space. Furthermore, as a consequence of universal aspects of physical spatial experience, a wide range of metaphorical correlations, including those connecting time and space, have been found to surface across genetically unrelated languages and cultures (Haspelmath, 1997; Moore, 2017; Duffy and Feist, 2023). For these reasons, metaphors drawing upon the source domain of space and, more specifically, the connections between space and time, have provided a rich test bed for investigating claims about the complex nature of metaphorical thinking.

As many have noted, however, linguistic evidence alone is insufficient to establish the conceptual nature of metaphoric connections (Murphy, 1996; McGlone, 2001; Gibbs and Perlman, 2006), leading researchers to seek nonlinguistic evidence for these connections. In tests of the psychological reality of the connections underlying spatial metaphors for time, particular attention has been paid to two deictic spatial metaphors that are used in the conceptualization of time: the Moving Ego metaphor and the Moving Time metaphor. The Moving Ego metaphor construes time as a stationary landscape that the active ego moves across—as evidenced in expressions such as “We’ve *arrived* at the moment of truth” (referenced above) and “We’ve *passed* the deadline.” In contrast, the Moving Time metaphor represents time as a conveyor belt on which events move, from the future to the past, relative to a stationary ego—as shown in expressions such as “Spring is *coming*” (referenced above) and “The election is *approaching*.” In addition to linguistic evidence for these space–time metaphors, a variety of studies have provided evidence of their psychological reality. To begin, McGlone and Harding (1998) found that there is a cognitive cost associated with switching between the Moving Ego and Moving Time metaphors, suggesting that they rely on different underlying conceptual structures. Building on this research, Boroditsky (2000) and Boroditsky and Ramscar (2002) asked whether thinking about spatial motion either toward or away from the self can prime construals of time consistent with the direction of motion, as would be expected if these metaphors truly draw upon spatial conceptualizations. When their participants responded to McGlone and Harding’s (1998) ambiguous experimental probe, *Next Wednesday’s meeting has been moved forward two days*. *What day is the meeting now that it has been rescheduled?* those who had been primed with a self-motion scenario—for instance, moving through space toward a stationary object (in line with the Moving Ego perspective)—were more likely to re-use this perspective for time and answer *Friday*. By contrast, when participants imagined motion toward the self—such as imagining a moving object traveling through space toward them (in line with the Moving Time perspective)—they were more likely to respond *Monday*. Based on these findings, Boroditsky and Ramscar (2002, *cf*., Boroditsky, 2000) argued that people’s thinking about time is closely linked to their spatial experiences, such that engaging in thought about motion in space can influence how people reason about time. Subsequent research conducted in a range of settings has provided further support for this conclusion, with demonstrations that non-deictic spatial schemas (Kranjec, 2006; Núñez et al., 2006), abstract spatial motion schemas (Matlock et al., 2011), fictive motion schemas (Matlock et al., 2005; Ramscar et al., 2010), and gesture (Jamalian and Tversky, 2012; Lewis and Stickles, 2017; Winter and Duffy, 2020)—all of which draw upon spatial thinking—may similarly influence how people think about time.

However, while there are indeed universal, shared bodily experiences that can provide a motivation for our metaphorical understanding of abstract concepts, a sole focus on those shared experiences leaves gaps in our understanding of the workings of metaphor. First, although physical spatial experiences may be similar around the world, linguistic evidence suggests that spatial experiences may not be conceptualized in exactly the same way in different cultures (e.g., Pederson et al., 1998; Majid et al., 2004; Feist and Zhang, 2019). It is therefore an open question whether cross-cultural differences in the conceptualization of physical space may give rise to concomitant differences in metaphorical conceptualizations that draw upon space. Furthermore, on an individual level, physical and situational differences may result in variation in people’s lived experiences and, thus, in the ways in which people talk and think about the world around them (Gibbs, 2009, 2011). As a result, individual variation in such factors as age, gender, body size, and physical (dis)ability, as well as less tangible factors such as ideology, religious beliefs, and culture, may thereby impact the ways in which speakers conceptualize both their physical and their metaphorical experiences (Dąbrowska, 2016; El Refaie, 2019; Littlemore, 2019).^1^ In this way, a complete understanding of metaphor must extend beyond the influences of universal bodily experience, including potentially universal experiences of space, to seek out the roles that may be played by each of these more individual factors.

Indeed, recent lines of empirical research have begun to yield evidence that metaphors are understood differently by different people. For instance, gender may play a role in influencing people’s perceptions of power metaphors, with one study finding that male participants were faster to identify powerful groups when they were labeled as male and presented at the top of a computer screen (Winter et al., 2020; see also Charteris-Black, 2012; Pérez-Sobrino et al., 2021). Handedness may also play a role in metaphoric reasoning: right-handers have been found to make more favorable judgments about objects that are presented on their right-hand side, while left-handers have been found to display more favorable judgments about those presented on their left-hand side (Casasanto, 2009a; see also Casasanto and Jasmin, 2010; Casasanto and Henetz, 2012; but see Sasaki et al., 2019 for contrasting findings in Japanese). Other research has found that metaphorical mappings of morality in space are more prominent among those practicing religion than among atheists (Li and Cao, 2017), suggesting that religious beliefs may likewise play a role in metaphor interpretation.

With evidence that individual factors play a role in the use and interpretation of metaphor accumulating, the goal of the current review article is to seek a clearer understanding of this role through a focused examination of individual factors in one domain. In recent years, the domain of time, along with its metaphorical connections to space, has offered the “model system of choice” for linguistic and psychological investigations into the relationships between metaphorical source and target domains (Casasanto, 2009b, p. 128). For this reason, the current review will draw upon spatial metaphors for time as a test bed to seek out the range of ways in which a person’s individual experience of the world may, in turn, color their interpretation of metaphor. Through an examination of the ways in which the domain of space may interact with an individual’s embodied and enculturated personal experiences to give rise to a metaphorical conceptualization of time, we aim to likewise expand understanding of the inner workings of metaphor broadly construed.

## 2. Time and space across languages

Spatial metaphors for time are used in a wide variety of languages around the world (Traugott, 1978; Haspelmath, 1997; Yu, 1998; Núñez and Sweetser, 2006; Filipović and Jaszczolt, 2012; Moore, 2014; Duffy and Feist, 2023; *inter alia*). However, while McGlone and Harding’s (1998) *Next Wednesday’ meeting* question has provided an ingenious way to probe the conceptual connections between space and time, cross-linguistic investigations reveal that the ambiguity observed in English may not emerge in all languages. Speakers of Swedish, for instance, demonstrated proclivity (over 80%) for the Moving Ego perspective in both primed and unprimed contexts (Rothe-Wulf et al., 2015), whereas speakers of Mandarin Chinese were found to be more likely (over 85%) to adopt the Moving Time perspective (e.g., Bender et al., 2010; Li, 2020). Within a language as well, we find that speakers from different communities respond differently to translations of this probe: although Belgian speakers of Dutch tended to adopt the Moving Ego and Moving Time perspectives at chance levels in response to the construction *voorwaarts verplaatst* (moved forward; Elvevåg et al., 2011), speakers of Dutch from the Netherlands tended to prefer the Moving Time perspective in response to another translation of the probe, *naar voren verplaatst* (moved forward; 74%; Loermans et al., 2019). In parallel fashion, speakers of Standard German interpreted the *Next Wednesday’s meeting* probe in line with the Moving Time perspective up to 95% of the time (Bender et al., 2005, 2010; Rothe-Wulf et al., 2015), while speakers of Swiss German evidenced different interpretations depending on how the probe was translated (Stocker and Hartmann, 2019). In response to translations using the construction *nach* *für*e *verschobe* (moved forward), both *Monday* and *Friday* responses were elicited (59% and 41%, respectively), but in response to translations using the construction *nach* *vor**ne* *verschobe* (moved forward), Swiss German participants (similarly to Standard German participants) showed a reliable preference for the Moving Time perspective (89%). The particulars of the translation likewise influenced interpretations among speakers of Mandarin Chinese. Whereas Li (2020) found that Mandarin speakers overwhelmingly interpreted *qian yidong* (forward move) in line with the Moving Time perspective (87% *Monday* responses), he found that a different version of the question that simply utilized *yidong* (move) was more ambiguous, with 41% of his participants adopting the Moving Time perspective and 59%, the Moving Ego perspective.^2^

Looking beyond the *Next Wednesday’s meeting* paradigm, the mapping of *earlier* and *later* times to the sagittal axis^3^ (front-back) in naturally occurring metaphors varies across the languages of the world. When Ego is involved, as in the Moving Ego and Moving Time metaphors that play a role in the *Next Wednesday’s meeting* probe, we find metaphors in which the future is mapped to Ego’s *front* in English, as well as in Japanese (Shinohara and Pardeshi, 2011; Moore, 2014), Wolof (Moore, 2014), Mandarin Chinese (in limited uses; see Yu, 1998; Ahrens and Huang, 2002; Feist and Shi, n.d.), Danish Sign Language (Engberg-Pedersen, 1993), and American Sign Language (Emmorey, 2001). A more limited set of languages has been found that map the future in these metaphors to Ego’s *back*, including Aymara (Núñez and Sweetser, 2006), Vietnamese (Sullivan and Bui, 2016), and Mandarin Chinese (Alverson, 1994; Feist and Shi, n.d.). For metaphors in which Ego is not involved (i.e., when times are located relative to other times), mappings to the sagittal axis suggest a direction of motion attributed to time or an event, similar to that implicated in the Moving Time metaphor. This direction likewise varies across languages: earlier times are *in front of* later ones in English, Wolof (Moore, 2014), Japanese (Shinmura, 1998, cited in Moore, 2014), Vietnamese (Sullivan and Bui, 2016), Yucatec Maya (Le Guen and Pool Balam, 2012), Aymara (Núñez and Sweetser, 2006), Mandarin Chinese (Yu, 2012; Feist and Shi, n.d.), Tzeltal (Brown, 2012), and Yupno (Cooperrider et al., 2022). In contrast, Hausa orders events with *earlier* in front of *later*, but days with *later* in front of *earlier* (Hill, 1978); the *later* in front of *earlier* mapping is also observed in Japanese and Marathi (Shinohara and Pardeshi, 2011).

Taken together, these findings suggest that, although the Moving Time and Moving Ego metaphors and the mappings underlying them are attested across a range of languages, there is no single, universal temporal structure that is being mapped, even within a single language. This variation thus gives rise to questions about whether there may be other factors, in addition to language, which influence the mapping from space to time. It is to those questions that we now turn, beginning with an exploration of factors that relate to the individual cognizer before expanding our examination to include factors related to the temporal entity being described and the context within which the metaphor is used.

## 3. Homing in on the individual

Building on evidence that the Moving Ego and Moving Time metaphors are psychologically real (McGlone and Harding, 1998) and, further, that they draw upon conceptual connections between time and space (Boroditsky, 2000; Boroditsky and Ramscar, 2002), a variety of studies have sought to further illuminate the structuring of time as a linear entity ordered along a sagittal axis. As we will see, in addition to strengthening the evidence for a conceptual connection between space and time, these studies have uncovered evidence that the connection is more intricate than that suggested in Conceptual Metaphor Theory (e.g., Lakoff and Johnson, 1980; see Duffy and Feist, 2023).

### 3.1. Approach and avoidance

In addition to time, a variety of emotions and personality traits have been argued to be connected to space and, more specifically, to spatial approach and avoidance, including anger, happiness, and extroversion. In space, approach entails movement in a forward direction along the sagittal axis, while avoidance may be connected to either backward movement along this axis or stasis. The similarities between these motions and those underlying the Moving Ego and Moving Time metaphors have inspired the examination of connections between approach- and avoidance-related factors and temporal metaphors.

For example, based on its associations with approach-related motivations, Hauser et al. (2009) hypothesized that a shared approach-related spatial motivation might serve as an embodied cognitive link between anger and the Moving Ego representation of time. To test for this connection, in one experiment, Hauser et al. (2009) asked English-speaking participants to complete a series of questionnaires designed to measure trait anger (that is, anger as part of their personality) before answering the *Next Wednesday’s meeting* question. As predicted, the findings revealed that participants who averaged higher trait anger were more likely to respond *Friday* (consistent with the Moving Ego perspective) than to respond *Monday* (consistent with the Moving Time perspective).

Examining this relationship further, Hauser et al. (2009) next sought evidence of a potential bi-directional relationship between the two domains, asking whether manipulating representations of time could affect feelings of anger, much in the way that feelings of anger affect reasoning about time. Using a scheduling task designed to prime either the Moving Ego or Moving Time perspective, they found that, when asked to rate how angry they were feeling at the current moment, participants in the Moving Ego condition reported significantly higher scores for reported anger than did participants in the Moving Time condition. Taken together, these findings suggest that the metaphorical domains of anger and time appear to influence one another in a bi-directional manner, in line with the correlations observed between temporal perspective and approach and avoidance motivations.

Looking to other approach- and avoidance-related factors, Richmond et al. (2012) sought to investigate a connection between emotional state (e.g., happy, sad, anxious) and temporal perspective. Reasoning that feeling in control and proactively approaching a positive future is more likely to be connected to positive feelings such as happiness, Richmond et al. (2012) hypothesized that people who adopt the Moving Ego perspective (responding *Friday*) would report experiencing significantly higher levels of happiness. By contrast, passively awaiting the arrival of events is more likely to be connected to negative feelings; hence, people who adopt the Moving Time perspective (responding *Monday*) would report experiencing significantly higher levels of anxiety and depression. As predicted, when they compared British participants’ responses to the *Next Wednesday’s meeting* question with their scores on a series of questionnaires for assessing individual differences in levels of happiness, anxiety, and depression, they found that participants who responded in line with the Moving Ego perspective likewise reported higher levels of happiness, while participants who responded in line with the Moving Time perspective reported higher levels of anxiety and depression.

Like Hauser et al. (2009), Richmond et al. (2012) next investigated the question of whether people’s perspectives on time could influence their emotional experiences. They found that after completing a task designed to prime either the Moving Ego or Moving Time perspective through rescheduling a series of events on a timeline (*cf.*, Hauser et al., 2009), English-speaking participants in the Moving Ego condition self-reported higher levels of happiness, while those in the Moving Time condition self-reported higher scores for anxiety and depression, thus revealing a bi-directional link between approach- and avoidance-related emotional experiences and perspectives on time.

Looking beyond emotion, one of the most fundamental dimensions of personality, extroversion (e.g., Eysenck, 1947; Briggs-Myers and Briggs, 1985; Costa and McCrae, 1985; John, 1990), is likewise grounded in approach motivations. Extroverted individuals are characterized as assertive and sociable, with the main direction of their energies oriented toward the outer world of material objects and people (John, 1990; John and Srivastava, 1999; John et al., 2008)—much in the way that in the Moving Ego metaphor, the self actively approaches events in the future. By contrast, introverted individuals are characterized as reserved and withdrawn, exhibiting a more passive perspective on the social and material world (John, 1990; John and Srivastava, 1999; John et al., 2008)—much in the way that in the Moving Time metaphor, the self passively awaits the arrival of events. Given the connections between other approach- and avoidance-related traits and temporal perspective reviewed above, we hypothesized that there would be differences in temporal reasoning between extroverts and introverts that parallel the spatially-based alignment of these personality types with approach and avoidance, with extroverts displaying more of a tendency to view themselves as approaching future events and introverts showing a greater likelihood of viewing future events as approaching themselves (Duffy and Feist, 2014). To test this, we had British participants complete a questionnaire for measuring extroversion-introversion (BFI; John, 1990) before responding to the *Next Wednesday’s meeting* question. As anticipated, participants who adopted the Moving Ego perspective (answering *Friday*) exhibited higher degrees of extroversion compared to participants who adopted the Moving Time perspective (responding *Monday*), suggesting that the shared spatial motivations may create a link whereby metaphorical reasoning is influenced by not only emotion, but also personality factors.

Noting that the valence of a given social event, and the concomitant desire to approach or avoid it, may differ for different personality types, Duffy and Evans (2017) reasoned that a question about moving *next Wednesday’s party* should result in a stronger extroversion effect than that in the original Duffy and Feist (2014) finding. To test this possibility, they presented British participants with a version of the *Next Wednesday* probe that replaced *meeting* with *party*, along with the eight extroversion statements from the BFI (John, 1990). In contrast to the original finding featuring a neutral event (Duffy and Feist, 2014), however, Duffy and Evans (2017) found that *Monday* and *Friday* responders did not differ in their extroversion/introversion scores, giving rise to the possibility that the individual factors influencing metaphor interpretation are interactive rather than additive.

### 3.2. Lifestyle and control over time

Alongside connections to approach and avoidance, the Moving Ego and Moving Time metaphors differ with regards to the amount of inferred control that Ego has relative to Time. Noting that the majority of studies using the *Next Wednesday’s meeting* question had sampled student populations, but that the lifestyle of a student is not representative of the general population, we investigated whether lifestyle may also influence an individual’s approach to time and resulting resolution of a temporally ambiguous statement. One salient difference between full-time employees and students is that, whereas workers are paid for their time and have little choice about whether or when they will work, students pay to attend university. As the consumers, students typically have the choice of whether or not to turn up to a lecture, and when to work on their course-related tasks. The flexibility of time inherent in the student lifestyle thus stands in stark contrast to the more rigid structure of time inherent within full-time employment. In light of these differences, and in view of insights from Richmond et al. (2012), who found that English speakers who report higher levels of perceived personal agency were more likely to adopt the Moving Ego perspective, we hypothesized that people who have greater control over the structuring of their time and more temporal flexibility in their daily lives, such as students, may view time differently and, thus, may interpret temporal metaphor differently from those who are subject to more external constraints and who require higher degrees of time management in their professional lives, such as administrators (Duffy and Feist, 2014). To test this hypothesis, we presented the ambiguous *Next Wednesday’s meeting* question to UK-based university students and to administrators (such as personal assistants, secretaries, and university timetable coordinators) who are tasked with the daily management of a multitude of events and activities. As anticipated, the findings revealed a difference between the two groups, with administrators more frequently adopting the Moving Time perspective (responding *Monday*; 71%) and students, the Moving Ego perspective (responding *Friday*; 61%).

Similarly to lifestyle, religion may also influence the extent to which a person believes themself to have control over their life. Central to Taoism is the practice of *wu-wei*—that is, the action of non-action (Maspero, 1981; Loy, 1985); thus, Li and Cao (2021) hypothesized that Taoists would be more likely to view time as approaching themselves (in line with the Moving Time perspective) than would their non-religious counterparts. These predictions were supported by participants’ responses to a Mandarin Chinese version of the *Next Wednesday’s meeting* question (*cf.*, Zheng et al., 2019), providing additional evidence for the connection between feelings of control and people’s metaphorical perspectives on time.

More directly addressing the question of control, Loermans et al. (2019) invited speakers of English and Dutch to think of a situation in which they were (or were not) in control before responding to the *Next Wednesday’s meeting* probe. While they found a connection between feelings of control and responses to the *Next Wednesday’s meeting* probe among English-speaking participants^4^ (*cf.* Richmond et al., 2012), this connection was not observed among Dutch-speaking participants^5^ (but see Mikša et al., 2018 for evidence of this connection in Croatian). Moreover, a follow-up study revealed that Dutch speakers who responded using the Moving Ego perspective (a minority of their participants, at 24%) and those who responded using the Moving Time perspective (the majority of their participants, at 74%) did not differ in terms of how much control they reported feeling over events in their lives.^6^ Taken together, these findings suggest that the roles of individual factors, such as feelings of control, may be intricately tied to cultural factors, such that no single factor influences metaphor interpretation in isolation.

Control may be connected to approach and avoidance motivations, as well. As seen in section 3.1, people with a variety of personality traits associated with approach motivations tended to favor the Moving Ego perspective, while those who embodied personality traits associated with avoidance motivations tended instead to favor the Moving Time perspective. This correlation raises the question of whether encouraging people to experience approach-motivated feelings might give rise to a preference for the Moving Ego perspective (and vice versa for discouraging these feelings). Similarly to other approach-motivated traits, feelings of power have been argued to trigger disinhibited behavior and a sense of control over the environment, while powerlessness triggers “those features of the self-relevant to others’ goals” (Keltner et al., 2003, p. 265), aligning well with the Moving Ego and Moving Time perspectives, respectively. However, in contrast to other approach-motivated traits, feelings of power may be induced experimentally in the lab (Carney et al., 2010), thus allowing for the connection between power and temporal perspective to be examined independent of other contributing personality factors.

Like other animals, humans express high levels of power spatially through expansive and open postures, which maximize the use of occupied space, whereas low levels of power are conveyed through contractive and closed postures, which minimize the use of occupied space (Darwin, 1872/2009; de Waal, 1998; Carney et al., 2005). Research has shown that maintaining high-power and low-power poses may not only reflect feelings of power, but also produce them (Carney et al., 2005, 2010; Cuddy et al., 2015; Ranehill et al., 2015), even after as little as 2 min of adopting the pose (e.g., Carney et al., 2010). Building on these findings, we sought to investigate whether the effects of enacting a high- vs. low-power pose might extend to temporal reasoning due to the embodied cognitive link between power and time via the shared intersections between the two domains with approach and avoidance motivations (Duffy and Feist, 2017).

Across two temporal tasks, we found that undergraduate student participants from the UK who adopted high-power poses demonstrated a greater preference for the Moving Ego perspective, compared to those adopting low-power poses. Notably, however, the effects of enacting a high-power pose appear to be stronger than the effects of enacting a low-power pose. First, participants who enacted a high-power pose responded to the *Next Wednesday’s meeting* question with *Friday* more often than with *Monday*. By contrast, participants enacting low-power poses demonstrated no preference for either response. Second, when indicating a preference between syntactic framing for temporal metaphors consistent with the Moving Ego perspective (i.e., *We’re approaching Christmas*) and framing consistent with the Moving Time perspective (i.e., *Christmas is approaching*), participants in the high-power pose condition preferred statements consistent with Moving Ego while participants in the low-power pose condition preferred statements consistent with Moving Time, as predicted. However, the preference displayed by the high-power pose participants was greater than that displayed by the low-power pose participants.

This asymmetry may shed further light on details of the spatial motivations underlying the metaphorical connections. First, as mentioned above, approach motivation is typically associated with forward movement of the self, while avoidance motivation tends to be connected to backward movement (e.g., Carver and Scheier, 1998; Elliot, 2006). Studies examining connections between imagined movement and metaphorical temporal reasoning have revealed that these two directions are not symmetrical: whereas participants primed with abstract forward motion (i.e., progression along a series, such as counting up or imagining letters of the alphabet in order) were more likely to respond *Friday* to the *Next Wednesday’s meeting* question, in line with the Moving Ego perspective, participants primed with abstract backward motion sequences (i.e., counting down or imagining letters of the alphabet in reverse order) showed no preference for *Friday* vs. *Monday* responses (Matlock et al., 2011). One explanation for this pattern of effects lies in the fact that, while forward and backward motion involve symmetric directions, the two directions are not equally frequent in everyday experience (Matlock et al., 2011), thereby blunting the influence of the less frequent direction.

In addition, the two directions of motion are not equally strongly associated with approach and avoidance motivations, respectively (Duffy and Feist, 2017). Specifically, although approach motivations have consistently been associated with active, forward motion, avoidance motivations have been connected to two different kinds of behaviors: in addition to backward motion, avoidance motivations have been connected to passive behaviors, which do not imply motion (*cf.*, Richmond et al., 2012). For this reason, approach motivations may be more strongly connected to forward motion than avoidance motivations are to backward motion, giving rise to stronger effects of high power via the links between level of power, spatial motivation, and temporal perspective.

### 3.3. Procrastination and conscientiousness

Although the contrast in temporal perspective between students and administrators noted in Section 3.2 may stem from a difference in terms of control over time and time management demands, there are additional differences between the two groups that may be connected to temporal perspective. For example, research shows that procrastination is particularly common in the academic domain, with up to 95% of students habitually deferring academic tasks such as writing assignments, studying for examinations and keeping up-to-date with weekly seminar reading (Ellis and Knaus, 1977; see also Solomon and Rothblum, 1984; Ferrari and Beck, 1998). In contrast to the student population, procrastination has been found to chronically affect only 15%–20% of nonstudent adults, with the lowest rates of procrastination reported by professional, business, and educational employees, such as university administrators (Harriott and Ferrari, 1996). In line with these norms, criteria such as “excellent organizational skills” and the “ability to prioritize workload and manage conflicting priorities” commonly feature in job advertisements for university administrators (*cf.*, Work4Northumbria, 2022).

Procrastination involves the movement of tasks “forward” into the future, in a direction defined by the ego’s movement through time (concordant with the Moving Ego perspective), while prioritization (associated with conscientiousness) entails the movement of tasks “forward” toward the present, ergo toward the ego (concordant with the Moving Time perspective). Thus, we hypothesized that the habitual movement of tasks in one of these directions may be a contributor to the temporal perspective adopted in response to the *Next Wednesday’s meeting* question, with procrastinators favoring the Moving Ego perspective, and conscientious individuals favoring the Moving Time perspective (Duffy and Feist, 2014). To test this hypothesis, we asked undergraduate students in the UK to complete a questionnaire for measuring trait procrastination (Lay, 1986) and trait conscientiousness (John, 1990) before they provided a response to the *Next Wednesday’s meeting* question. Consistent with our predictions, we observed a significant effect, with participants who adopted the Moving Ego perspective (answering *Friday*) reporting higher procrastination scores and lower conscientiousness scores than participants who adopted the Moving Time perspective (answering *Monday*), thus expanding the growing list of personality factors that may be connected to temporal perspective and the interpretation of ambiguous metaphoric language.

Noting that these results, like those from other studies investigating the effects of personality differences on metaphorical temporal reasoning, have relied on participants’ self-reported assessments of the variables under study, we next set out to test whether these relationships can similarly be observed under real world conditions. To do this, we turned our focus to real-life timeliness behaviors that correlate with procrastination and conscientiousness, comparing the resolution of temporal ambiguity to the timeliness of: (1) workers traveling to work; (2) students submitting an essay on campus; and (3) people arriving for a scheduled appointment. Across three experiments, we found that UK-based participants who adopted the Moving Ego perspective were more likely to procrastinate, meeting their obligations later on average than participants who adopted the Moving Time perspective, thus extending earlier findings with evidence that these effects reach beyond the laboratory.

Much like procrastination, a person’s “body clock,” i.e., their chronotype, likewise represents differences in people’s temporal preferences, with some people preferring evenings and others, mornings. In addition, earlier lines of research have shown an association between a preference for evenings and procrastination (Ferrari et al., 1997; Przepiórka et al., 2019), suggesting that chronotype, like a tendency to procrastinate (or not to do so), may influence how people reason about ambiguous temporal metaphors. Building on findings suggesting a connection between temporal perspective and procrastination (Duffy and Feist, 2014; Duffy et al., 2014), Shen and Li (2021) administered a Morningness–Eveningness Questionnaire (Horne and Ostberg, 1976) along with the *Next Wednesday’s meeting* question to Chinese university students to examine the relationship between choronotype and temporal perspective. Their findings showed that those who adopted the Moving Ego perspective displayed greater preferences for evening over morning, while those who adopted the Moving Time perspective displayed the opposite preference, concordant with the connections between procrastination and temporal perspective observed in prior research (Duffy and Feist, 2014; Duffy et al., 2014).

Throughout Section 3, we have seen that the interpretation of space–time metaphor is influenced by a variety of individual factors in addition to spatial experience, suggesting that metaphor involves additional factors beyond the mapping from a source domain to a target domain. One common thread running through these factors is their connection to either spatial or temporal experience, suggestive of an interconnected conceptual network underlying the mappings that surface in metaphor. Because cognition exists not only within the mind, but also in the systems with which we interact (Hutchins, 1996), these findings of individual variation in the interpretation of metaphor raise questions about whether parallel factors associated with the context of use of a space–time metaphor may likewise influence metaphor interpretation.

## 4. The contextualized individual

Just as individuals differ in complex ways, so do the events they describe and the situations in which they speak. For example, speakers may discuss the timing of emotionally charged events, thereby opening a pathway for the valence of the event to impinge upon temporal reasoning (*cf.*, section 3.1). Speakers may also reason about time across a range of contexts, including contexts that mimic the longer-term time pressures that characterize different lifestyles (*cf.*, section 3.2). These connections between the individual-level factors surveyed in Section 3 and the broader contexts within which speakers communicate raise questions about whether the nature of the event, as well as contextual factors, may likewise exert an influence on metaphoric interpretation.

### 4.1. Affect and features of the “meeting”

As with individuals, there is wide variation in the range of events that people may schedule and reschedule. Noting that McGlone and Harding’s (1998) ambiguous question asks about the rescheduling of a “meeting,” the nature of which is unspecified to the comprehender, one question that has been raised is whether the valence of the event being moved (positive or negative) may influence whether people consider themselves to be approaching the event or the event to be approaching them. Reasoning that positive affect tends to be spatially represented by approach motivations and negative affect, by avoidance motivations (Cacioppo et al., 1993; Chen and Bargh, 1999; Neumann et al., 2003;^7^ *cf.* section 3.1), Margolies and Crawford (2008) hypothesized that positively-valenced events might encourage use of the Moving Ego perspective and negatively-valenced events might encourage use of the Moving Time perspective. To test this hypothesis, they asked participants from the US to envisage an event, scheduled for “next Wednesday,” for which they might feel either enthusiasm (e.g., seeing a distant loved one) or dread (e.g., a stressful exam). Participants then responded to a series of task-related questions about the new timing for the event and how they felt about its rescheduling, including both the *Next Wednesday’s meeting* probe and a question directed at whether they felt themselves to be approaching the event or the event to be approaching them. As predicted, Margolies and Crawford (2008) observed an association between positive event valence and the Moving Ego perspective and, conversely, between negative event valence and the Moving Time perspective, with participants who responded to a positively-valenced event being more likely to describe themselves as approaching the event, and participants who responded to a negatively-valenced event being more likely to describe the event as approaching themselves. However, the valence of the event did not significantly affect participants’ responses to the *Next Wednesday’s meeting* question itself (i.e., whether they responded *Monday* or *Friday*); an apparent inconsistency that may stem from a tension between the tendency for people to imagine moving toward positive events (in line with the Moving Ego perspective) and people’s desire for positive events to occur sooner (resulting in a direction of change that aligns with the Moving Time perspective). Building on these findings in a follow-up study, Margolies and Crawford (2008) found that participants judged an event to be more positive when it was described using the language of Moving Ego compared to the language of Moving Time, thus suggesting that space–time metaphors also convey information about the valence of an event^8^ and strengthening the evidence in support of a bi-directional link between metaphor and individual factors. In discussing the implications of their findings, Margolies and Crawford concluded that:

Our embodied knowledge and perceptions are a result of an accumulation of sensorimotor experiences, including emotional reactions and spatial movements that influence each other in shaping thought. Abstract thought capitalizes on more concrete domains and thus is subject to influences from both physical and affective experience (2008, p. 1412).

The interrelation between event valence and metaphorical perspectives on time may also be seen in the ways in which speakers choose to describe the events that they have experienced. As a case in point, McGlone and Pfiester (2009) sought to examine whether people make differential use of Moving Ego and Moving Time when they communicate about positive and negative events. In this study, McGlone and Pfiester (2009) asked undergraduate participants in the US to write narratives about pleasant or unpleasant events that they had experienced in the recent past and to rate the events’ pleasantness on a scale. An analysis of the metaphorical temporal descriptions revealed that participants tended to use the Moving Ego perspective more frequently for describing pleasant events, e.g., “it was great hanging out in the blind and *passing the time* drinking beer” (p. 17), while the Moving Time perspective was employed more frequently for describing unpleasant events, e.g., “practically an *entire hour passed* while I just sat there” (p. 17). In addition, their findings suggest that speakers not only differentially employ these two perspectives in their descriptions of events, but also infer meaning from another’s use of these temporal perspectives. Thus, when participants were asked to read a fictitious first-person account of a journal entry describing the activities of a student over the course of a week during the academic year, they rated the narrator as more excited in contexts that utilized the Moving Ego metaphor and more worried in contexts that utilized Moving Time.

Turning from the lab to the real world, McGlone and Pfiester (2009) next examined the connection between affect and temporal metaphor in natural language uses of Moving Ego and Moving Time metaphors. Specifically, using a selection of American English corpora, they found that the valence of the encoded event (positive, negative, or neutral) co-varied with the temporal perspective adopted in the description. In line with the experimental results, positive events were more frequently encoded by the Moving Ego perspective, e.g., “There is much optimism that we might be *coming to*” (WordBanks USBooks Corpus, cited in McGlone and Pfiester, 2009, p. 13), while negative events tended to be encoded by the Moving Time perspective, e.g., “when *the time comes* she cannot do things and she has to be cared for” (Switchboard Corpus, cited in McGlone and Pfiester, 2009, p. 13), suggesting that the connections uncovered in laboratory experiments extend to natural uses of language.

Event valence has been observed to exert an effect on metaphorical temporal perspectives in Mandarin Chinese as well. For instance, in one study, speakers of Mandarin Chinese were primed with a scenario of a future event that was designed to elicit one of three emotions: happiness, anger, or anxiety (Zheng et al., 2019). They then responded to two measures of temporal perspective: an adapted version of the *Next Wednesday’s meeting* question, which replaced the meeting with the described event, and a time motion schema question (*cf.*, Boroditsky, 2000), depicting the Moving Ego and Moving Time perspectives, which asked participants to indicate whether they were approaching the event or whether the event was approaching them (*cf.*, Margolies and Crawford, 2008). Concordant with the findings from English, Zheng et al. (2019) found that participants primed with positively-valenced future events were significantly more likely to adopt the Moving Ego perspective across both measures of temporal perspective.^9^

This pattern of findings has not, however, been replicated in all languages studied. For instance, Loermans et al. (2021) found that speakers of Dutch who were primed with a positive future event were no more likely to adopt the Moving Ego perspective than were those primed with a negative future event. In addition, despite demonstrating a strong overall preference for disambiguating the *Next Wednesday’s meeting* question in line with the Moving Time perspective, Dutch-speaking participants showed no preferred temporal perspective in their responses to the time motion schema, thus reiterating the notion that certain measures may tap into temporal perspective in different ways (as seen in the work of Margolies and Crawford, 2008, described above). What did influence participants’ temporal perspective in this study, however, was the mere fact of having explicitly thought about their emotional response before interpreting the *Next Wednesday’s meeting* question: Loermans et al. (2021) observed a higher rate of Moving Ego responses among participants who had rated their emotional response to the event first and a higher rate of Moving Time responses among those who had rated their emotional response second. In discussing the implications of their findings, Loermans et al. (2021) argued that introspecting about the event in question may have led to heightened feelings of agency, echoing the connection between heightened feelings of control and the Moving Ego perspective reviewed above (section 3.2).

When an event is in our future, movement will result in a diminution of the time until the event takes place. This diminution in time underlies the reasoning connecting event valence to temporal perspective in the studies reviewed thus far. However, this leaves open the question of how speakers will make use of the two perspectives when an event took place in the past, in which case movement will result in a lengthening of the time separating them from the event. Does event valence similarly influence temporal perspective when speakers are considering past events? To find out, Lee and Ji (2014) asked participants at a Canadian university to write about a past experience in which they had either felt rejected or embraced by their friends. They found that participants who wrote about an experience of being rejected were more likely to adopt the Moving Ego perspective (i.e., actively moving away from the past), whereas participants who wrote about an experience in which they felt embraced were more likely to adopt the Moving Time perspective (i.e., remaining closer to the past). In this way, participants metaphorically minimized their distance from pleasant experiences and maximized their distance from unpleasant experiences. These findings present an interesting parallel with the findings regarding approach and avoidance reviewed earlier, suggesting that not only temporal motion, but also preferences regarding temporal location may give rise to variation in the perspective adopted when interpreting metaphor.

### 4.2. Metaphor in context

All language use occurs in a context. Not only do individuals and the events about which they speak exhibit substantial variation, but the contexts of use do so as well. The findings that individual differences, as well as emotional states, influence metaphor interpretation suggest that contextual differences may likewise influence the in-the-moment interpretation of a metaphor. For example, the findings reviewed above suggest that individual differences related to internally imposed time pressure, including lifestyle and personality, exert influence on how people reason about events in time. These findings raise the question of whether externally imposed factors related to time pressure, such as pace of life, may similarly influence temporal reasoning and metaphor interpretation. For example, people who live in larger, more populous areas have been found to exhibit a general tendency to experience life at a faster pace, and to experience higher time pressures than do people who live in smaller, less populous areas (Bornstein, 1979; Levine and Bartlett, 1984; Garhammer, 2002). Building on these findings, Li and Cao (2019) asked whether the type of area people live in and the concomitant pace of life might influence an individual’s metaphorical approach to time. They reasoned that residents of faster-paced, densely populated cities would feel themselves to be more regimented by the clock, and therefore would be more likely to demonstrate a preference for the Moving Time perspective. By contrast, residents of slower-paced, less populated cities may experience less time pressure and a greater degree of temporal flexibility, thereby raising the likelihood that they will demonstrate a preference for the Moving Ego perspective. To test this hypothesis, Li and Cao (2019) asked residents of more populous cities (New York City, NY, United States; London, United Kingdom) and residents of less populous cities (Albuquerque, NM, United States; Lancaster, United Kingdom) to respond to the *Next Wednesday’s meeting* question. In line with their predictions, Li and Cao (2019) found that participants living in New York City and London were more likely to respond *Monday* (in line with the Moving Time perspective), while participants living in Albuquerque and Lancaster were more likely to respond *Friday* (in line with the Moving Ego perspective). These results, thus, suggest that environmental differences in time pressures, like individual differences in attitudes toward time, may influence people’s adopted perspectives when resolving an ambiguous metaphorical question about time.

External demands, however, change over time and across contexts, thus bringing into question whether an individual’s metaphorical temporal perspective may likewise vary across changing external demands. In a short-term longitudinal study aimed at addressing this issue, Loermans and Milfont (2018) compared the responses of undergraduate students from New Zealand to the *Next Wednesday’s meeting* question over a five-month period. They observed a small but reliable increase in the proportion of Moving Time perspectives over this time period, with participants being significantly more likely to adopt the Moving Time perspective, responding *Monday*, in July (*N* = 246, 75%) than in March (*N* = 224, 68%). This shift gives rise to the question of whether the two times of year might differ in some way that would be connected to a change in preference for the Moving Time perspective (Duffy and Feist, 2023). To find out, we consulted the academic calendar for the university where Loermans and Milfont’s (2018) study was conducted (Victoria University of Wellington, 2021). We found that the new academic year tends to begin in late February/early March—coinciding with the time of the participants’ first responses in the experiment. Temporal milestones such as the start of a new academic year may be associated with the “fresh start effect” (Dai et al., 2015), in which people’s mindsets—particularly with regard to their perspectives on time—may include greater openness to new goals or to meaningful changes to behavior. Because self-motivation and the drive to attain goals are characteristic of high-level agency (Vallacher and Wegner, 1989), which has likewise been found to relate to the Moving Ego perspective (Richmond et al., 2012), we reasoned that participants may have been exhibiting a more agentic, goal-oriented mindset and, thus, a more active approach toward time, at the beginning of the new academic year. By contrast, July tends to mark the start of the middle term and tends to follow the “mid-year break.” At this point in the academic year, many students may have established their routines and, thus, they may be less likely to be thinking about how to structure their time. Furthermore, the mid-year break follows a three-week assessment and examination period, so students’ mindsets may be affected by their recent pressure to complete assignments. When Duffy and Evans (2017) presented UK university students with the question, *Next Wednesday’s **assignment deadline** has been moved forward two days*. *What day has the event been rescheduled to?*, they found that their participants were more likely to adopt the Moving Time perspective (responding *Monday*), suggesting that this pressure may be related to the adoption of the Moving Time perspective. In addition, the external demands of assessments and examinations may lead students to feel more regimented, much like the external demands associated with living in a fast-paced, populous city did for Li and Cao’s (2019) participants. Thus, the change in responses between the first and second sessions in Loermans and Milfont’s (2018) study may have arisen due to a difference in the temporal context of the two sessions, with the first being more conducive to the Moving Ego perspective and the second, to the Moving Time perspective (Duffy and Feist, 2023).

Temporal context may include not only the months of the year, but also the days of the week. As observed by both Winter and Duffy (2020) and Medimorec (2022), this contextual factor also influences metaphorical temporal perspective, with English speakers from the US responding *Monday* more frequently when questioned on a Monday, and *Friday* more frequently when questioned on a Friday. Probing this effect further, Winter and Duffy (2020) found that asking the *Next Wednesday’s meeting* question on days closer to Monday and Friday results in progressively more *Monday* or *Friday* responses, respectively. While one possible explanation for these effects is priming—knowing that it is a Monday may help *Monday* to spring to mind more easily (*cf.*, Medimorec, 2022), there may be additional factors at play. For instance, as seen in sections 3.1 and 4.1, *Friday* responses tend to be associated with happiness and with positive affect, while *Monday* responses tend to be associated with depression and with negative affect. Given that associations for both mood and affect are more negative for Mondays and more positive for Fridays (Areni et al., 2011; Stone et al., 2012; Ellis et al., 2015), *Friday* responses elicited on a Friday may have resulted from positive affect, while *Monday* responses elicited on a Monday may have resulted from negative affect (Medimorec, 2022).

Other contextual factors may similarly tap into affect and, thereby, may influence people’s metaphorical perspectives on time. In studies examining connections between temporal perspective and the experience of grief and trauma, for instance, Ruscher (2011) found that individuals forecasted shorter periods of grief in response to a brief vignette about a woman whose young son had died which made use of a Moving Ego framing, as compared to a vignette which made use of a Moving Time framing (Ruscher, 2011; see also Ruscher, 2012; Turner et al., 2020; Pfaltz et al., 2021).^10^ Taken together, the results reviewed in Section 4 suggest that metaphorical perspectives may encourage, as well as result from, variation in affect surrounding a context and a topic under discussion, as was seen with the interplay observed between metaphorical perspective and individual emotional experiences surveyed in section 3.

## 5. Discussion

How do we reason about abstract concepts, given that we cannot see them or touch them? One prominent theory—CMT—suggests that we draw upon our knowledge of concrete concepts, which we can reason about more easily, in order to scaffold our understanding of abstract ones (Lakoff and Johnson, 1980). Conceptual metaphors thus represent an important cognitive tool whereby abstract concepts inherit structure from more concrete concepts (Lakoff and Johnson, 1980), resulting in both linguistic metaphors and patterns of thinking that bear the imprint of these metaphorical connections. Through the examination of people’s interpretations of an ambiguous temporal prompt, researchers in the cognitive sciences have uncovered compelling evidence that the metaphorical connections between space and time are not merely linguistic, but rather extend deep within the conceptual system. However, as the findings reviewed in the current article show, these connections alone fail to fully explain the use and interpretation of space–time metaphors. Rather, evidence is accumulating suggesting that the interpretation of metaphor is subject to a host of individual, event-based, and situational factors. This interplay of factors is what might be expected if, indeed, metaphorical connections extend throughout the conceptual system.

In the current review, we considered three types of factors: linguistic (and, relatedly, cultural) factors, individual factors, and contextual factors related to the content of the message and the situation within which it is spoken. At all three levels, we observed that, rather than resulting from a straightforward mapping from physical space to time, space–time metaphors bear the imprint of the individual speaker and the context within which she speaks. Taken together, this confluence of factors suggests that space–time metaphor is built up in real time rather than arising from either existing conceptualizations or a mapping between static domains.

Looking beyond the particular space–time metaphors that constituted the focus of the current review, we find that other metaphorical mappings from space to time are influenced by linguistic and cultural factors, in addition to interactions with other conceptual metaphors. Thus, for example, languages may make use of axes other than or in addition to the sagittal (e.g., Li, 2017 for Mandarin Chinese; Moore, 2014 for Wolof; and Brown, 2012 for Tzeltal); linear directionals may be rare or even absent in temporal metaphors (see Cooperrider et al., 2022, for Yupno; da Silva Sinha, 2019, for evidence from indigenous languages of Brazil); and the choice between one-dimensional and three-dimensional construals of temporal duration may be dependent on other contextually applicable metaphors for time (Alcaraz Carrión and Valenzuela, 2021).

Indeed, recent research in the cognitive sciences suggests that the interpretation of a variety of metaphors, like those describing time in terms of space, is influenced by multiple contextual and personal factors (Gibbs, 2009, 2017; Casasanto, 2017; Kövecses, 2017; El Refaie, 2019; Littlemore, 2019). This confluence of factors has led Littlemore (2019, p. 50) to argue that metaphor “varies according to the specific situations in which we find ourselves,” with those situations encompassing cultural and bodily circumstances alongside the context within which a metaphor is being used. In a similar fashion, Gibbs (2009, 2011, p. 552) highlighted the role of multiple, layered factors when he argued that metaphors may be “‘soft-assembled’ during thinking, speaking, and understanding.” At a broader level, Casasanto and his colleagues (Casasanto and Bottini, 2014; Casasanto, 2017; Pitt and Casasanto, 2020) argued for both high-level, general metaphoric links between domains and lower-level, more specific links, resulting in a layered set of influences on mental metaphors. Concordant with these proposals, we argue that the insight that metaphor involves a mapping from a source to a target domain is the beginning rather than the end of an account of the workings of metaphor. While important, this mapping is not accomplished in isolation. Rather, this mapping constitutes one set of inputs to a larger, complex, “blended” conceptualization (Fauconnier and Turner, 1998, 2002; *cf.*, Richards, 1936; Black, 1962), with metaphoric interpretation arising from the entire complex of domain-related factors, linguistic and cultural factors, individual factors, and contextual factors (Duffy and Feist, 2023). At a broader level, this blended conceptualization suggests a richly interconnected conceptual system whereby aspects of a variety of concepts may combine and recombine to create the range of in-the-moment interpretations observed in studies of space–time metaphor and in uses of metaphor in the real world.

## 6. Conclusion

In recent years, evidence across the cognitive sciences has revealed the importance of individual differences to the workings of the mind, demonstrating that, in order to piece together the puzzle of human cognition, we need to not only embrace this variation, but to integrate it with those findings which can be generalized (Prather et al., 2022; Scott-Phillips and Nettle, 2022). As we have seen, metaphor is no exception: research on the workings of metaphor has revealed a multiplicity of factors that together influence its use and interpretation, layered on top of the mapping from a source to a target posited by Conceptual Metaphor Theory. The variety uncovered, and the range of metaphoric mappings in which multiple factors have been observed, suggests that this work has just begun; the identification of additional factors, along with investigations of the ways in which these factors may interact with one another, will be necessary in order to fully understand the ways in which people draw upon one domain in service of understanding another.

Gibbs (2021, p. 494) points to a further challenge for metaphor researchers, noting that, while the use of metaphor is a general property shared by all humans, there are at the same time individual differences in the use and interpretation of metaphors, such that all people use metaphor in their own “unique, highly personal way.” As seen in the research surveyed in the current review, the factors influencing metaphor interpretation, like metaphor itself, include both general factors shared across communities and factors that are quite personal and individual. The influences of both general and individual factors on the interpretation of space–time metaphors thus suggest that a complete understanding of metaphor requires an understanding of both “the general within the specific and the specific within the general” (Gibbs, 2021, p. 496). The need to integrate these factors into a coherent, comprehensive account, therefore, points the way forward.

## Author contributions

MF and SD co-developed the argument and co-wrote the manuscript. Both authors approved the submitted version.

## Funding

The publication of this article was funded by Northumbria University’s Open Access Fund.

## Conflict of interest

The authors declare that the research was conducted in the absence of any commercial or financial relationships that could be construed as a potential conflict of interest.

## Publisher’s note

All claims expressed in this article are solely those of the authors and do not necessarily represent those of their affiliated organizations, or those of the publisher, the editors and the reviewers. Any product that may be evaluated in this article, or claim that may be made by its manufacturer, is not guaranteed or endorsed by the publisher.

## Footnotes

^1^See also Bergman and Lundh’s (2015) Editors’ introduction to the *Journal for Person-Oriented Research* for a broader perspective on the role of the individual in psychological science research.

^2^As Duffy and Feist (2023) note, along with cross-linguistic and cross-dialectal differences we find differences in the particular morphemes used in translations of the *Next Wednesday’s meeting* question across these studies, raising the possibility that variation in interpretations is driven by lexical choice (*cf.*, Feist and Duffy, 2015). Because there is no single ‘correct’ translation of any given sentence (*cf.*, Werner, 1995), however, these two variables are inextricably bound together, underscoring the role of the language one speaks in the final interpretation of a metaphor.

^3^This is the axis referenced in the *Next Wednesday’s meeting* question. Variation is likewise found in mappings which involve axes other than the sagittal (Duffy and Feist, 2023). For reasons of space, these variations will not be considered further here.

^4^However, English speakers displayed no preference between a Moving Ego framing (“I am approaching the meeting”) and a Moving Time framing (“the meeting is approaching”), reiterating the suggestion that different tasks may reveal different aspects of temporal perspective (Margolies and Crawford, 2008; see below).

^5^Moreover, a trend in the opposite direction was observed, with participants who were primed to think of a situation in which they were in control being more likely to adopt the Moving Time perspective in the *Next Wednesday’s meeting* question.

^6^As discussed above, however, the construction featured in the Dutch translation of the *Next Wednesday’s meeting* probe may not have been ambiguous, thus precluding clear conclusions concerning the absence of a connection between feelings of control and temporal perspective among speakers of Dutch.

^7^For contrasting findings, see Centerbar and Clore (2006) and Rotteveel et al. (2015).

^8^Noting that Monday and Friday differ in valence, with Friday generally being viewed more positively than Monday, in a follow-up study, Margolies and Crawford (2008) found that when the event was rescheduled to a less positive day (Monday) using a Moving Ego framing, participants still viewed the event more positively than when it was rescheduled to a more positive day (Thursday) using a Moving Time framing.

^9^It should be noted, however, that participants primed with negatively-valenced events also displayed a preference for the Moving Ego perspective, albeit to a significantly lesser extent.

^10^It should be noted, however, that other research has found that people primed with the Moving Ego perspective have underestimated to a greater extent the time needed to complete a task compared to those primed with the Moving Time perspective (Boltz and Yum, 2010), suggesting that people may underestimate durations of time that are framed from the Moving Ego perspective more generally.
